# Clinical Efficacy of CGF Combined With Periodic Supramolecular Salicylic Acid in the Treatment of Chloasma

**DOI:** 10.1111/jocd.70146

**Published:** 2025-03-26

**Authors:** Ge Su, Qiang Fu, Xun Zhou

**Affiliations:** ^1^ Department of Dermatology and Cosmetology Chongqing Hospital of Traditional Chinese Medicine Chongqing China

**Keywords:** CGF, efficacy, melasma, supramolecular salicylic acid

## Abstract

**Objective:**

To investigate the clinical efficacy of CGF combined with supramolecular salicylic acid cycle injection for melasma.

**Methods:**

Twenty patients with chloasma admitted to our hospital were selected and treated with CGF facial injection. After each CGF injection, supramolecular salicylic acid was given once. After one cycle of treatment, the severity of melasma and skin parameters before and after treatment were compared using the modified melasma Area and Severity index (MASI) and VISIA facial image analysis system, respectively. At the same time, the patients' self‐satisfaction was investigated.

**Results:**

The total effective rate of CGF combined with supramolecular salicylic acid in the treatment of chloasma was 95%. Compared with before treatment, the VISIA scores in spots, UV spots, brown spots, red areas, wrinkles, texture, pore and porphyrins scores were significantly increased after treatment. After CGF combined with supramolecular salicylic acid treatment, the MASI score of chloasma patients was significantly reduced (4.70 ± 2.47 vs. 15.30 ± 4.01, *p* < 0.001). The results showed that the total satisfaction of patients with facial skin improvement after CGF combined with supramolecular salicylic acid treatment was 95.00%.

**Conclusion:**

CGF combined with supramolecular salicylic acid has an obvious effect on the comprehensive overall improvement of melasma and facial aging patients, and can effectively accelerate the speed of skin lesion repair and pigment elimination, which is worthy of clinical promotion and application.

## 1 Introduction

Chloasma is a common pigmentary disorder skin disease that occurs in areas exposed to sunlight, mainly accumulating on the face, mostly symmetrically distributed on the cheeks, forehead, and chin. The lesions are butterfly shaped or diffuse light brown to dark brown patches with clear boundaries and varying sizes, most commonly found in women of childbearing age [1, 2]. Generally, there are no conscious symptoms, and the course of the disease is uncertain, which may last for several months or years [3]. The disease is stubborn and easy to recur, and often changes with the sun, emotions, sleep, and other factors [4]. The causes of chloasma are complex and varied, mainly genetic susceptibility and photoaging, while other causes include pregnancy, oral contraceptive pills, estrogen therapy, thyroid dysfunction, cosmetics, and phototoxic drugs and chronic diseases. The pathogenesis includes overactive melanocytes, inflammation, reactive oxygen species, skin barrier damage, increased vascularization, increased mast cells, upregulation of estrogen and estrogen receptors, abnormalities of the skin's extracellular matrix (ECM), and destruction of the basement membrane [5]. Pathological manifestations include increased melanin granules mainly in the basal layer and stratum spinosum, partly accompanied by increased melanin granules in the dermis [6]. Confocal laser scanning microscopy (RCM) images display bright and highly refracted melanocytes and granules in the epidermis; Rich and bright phagocytic melanocytes in the dermis [7]. Chloasma requires long‐term regular treatment. First of all, it is necessary to do a good job of daily sunscreen measures to avoid long‐term ultraviolet radiation‐induced aggravation of the symptoms and choose broad‐spectrum sunscreen with high SPF and PA values and moisturizing agents with barrier‐repairing effects to do basic care [8]. Conventional treatments include topical depigmentation agents, oral tranexamic acid, antioxidants, lasers, and chemical peels and other combined treatments [9, 10, 11]. However, there is no effective method to completely cure the disease. Chloasma and photoaging lesions have very similar pathologic features, and sun protection can significantly lighten the pigmentation, suggesting that photoaging also plays a very important role in the pathogenesis of melasma. Photoaging induces melasma formation by stimulating melanin synthesis through DNA damage [12]. In addition, photoaging leads to cellular senescence in the skin, and senescent cells stimulate melanin synthesis through the secretion of senescence‐associated secretory phenotypic molecules [13]. It has been shown that photoaging‐induced inflammation and its chemotactic inflammatory cells stimulate melanin synthesis [14]. Botulinum toxin injection is an effective treatment for photoaging of the skin with a wide range of clinical applications, while hyaluronic acid and autologous blood concentrates are also used in the treatment of photoaging [15].

Platelet‐rich plasma (PRP) and platelet‐rich fibrin (PRF) are first‐ and second‐generation derivatives of blood concentrates, respectively, and both contain a number of growth factors, including platelet‐derived growth factor (PDGF), insulin‐like growth factor (IGF‐1), transforming growth factor‐β1 (TGF‐β1), basic fibroblast growth factor (bFGF), vascular endothelial growth factor (VEGF) and epidermal growth factor (EGF), which promote tissue repair and regeneration [16, 17]. These platelet concentrates have recently been used in dermatology for tissue regeneration, wound healing, scar repair, skin rejuvenation, and hair loss. The growth factors in PRP stimulate the activation of fibroblasts, inducing the synthesis of collagen and other components of the extracellular matrix, which can reduce wrinkles and elasticity gain during the photo‐aging process of the skin [18].

Concentrated growth factor (CGF) is the latest blood concentrate [19]. CGF contained the same or higher levels of bFGF as PRF and activated PRP, while the levels of other growth factors were comparable in all three concentrates. Some findings have shown that CGF promotes the proliferation and activation of fibroblasts, thereby increasing the deposition of new collagen [20]. In addition, CGF facilitates the proliferation and differentiation of endothelial cells, leading to tissue neovascularization. Meanwhile, salicylic acid has been widely used as a bactericidal and anti‐inflammatory agent in the clinic. The study showed that acetylsalicylic acid induced morphological changes in melanocyte dendrites under UVB, thus exerting the function of decreasing melanosome transport [21]. Laser combined with salicylic acid in the treatment of chloasma has been reported. The new supramolecular salicylic acid, with its supramolecular slow‐controlled release delivery system, circumvents the instability and non‐water solubility of salicylic acid, which makes it less irritating and safer for the treatment of patients with sensitive and inflammatory dermatological diseases in the clinic. Regarding the treatment of melasma, there is no specific treatment available, and combination applications have been found to be effective in clinical practice. However, no study has yet reported the clinical efficacy of CGF combined with supramolecular salicylic acid in the treatment of melasma. In order to fill this research gap and confirm the improvement effect of CGF combined with supramolecular salicylic acid on skin condition, this study used CGF combined with supramolecular salicylic acid to perform superficial exfoliation on patients with chloasma and explored the therapeutic effect of this treatment method in the form of self‐control, so as to provide reference for clinical practice.

## 2 Materials and Methods

### 2.1 General Information About Patients

Patients with chloasma admitted to our hospital from May 2019 to March 2024 were selected as the research objects. Chloasma was diagnosed according to the Chinese Expert Consensus on the Diagnosis and Treatment of Chloasma (2021 edition). This study was approved by the ethics committee of Chongqing Hospital of Traditional Chinese Medicine, and all patients signed informed consent.

### 2.2 Inclusion and Exclusion Criteria

Inclusion criteria: (1) Patients met the diagnostic criteria for chloasma; (2) patients are willing to accept combined external facial treatment; (3) patients did not use other treatment methods in the first 6 months of treatment and during treatment; and (4) in the past 8–12 weeks, there was no significant UV exposure on the patient's face.

Exclusion criteria: (1) Pregnant women or patients in lactation; (2) patients with local skin inflammation or other skin diseases; (3) incomplete data may affect the diagnosis and efficacy evaluation of patients; (4) patients with chloasma in the past 6 months; (5) patients with severe heart, kidney, liver, endocrine and other systemic diseases, tumors, immunodeficiency diseases, and patients who have recently used special drugs such as antidepressants; and (6) patients with unrealistic expectations for treatment outcomes.

### 2.3 Methods

#### 2.3.1 Preparation of CGF

An appropriate amount of autologous blood was withdrawn from the patient and separated using a CGF centrifuge (MEDIFUGE CGF) and prepared by a laboratory biologist. After separation, the uppermost layer of platelet poor plasma (PPP) was collected using a 2.5 or 1 mL syringe. Autologous blood products rich in platelets and CD34+ cells (hematopoietic stem cells), the third generation of CGF, were extracted by heating at a constant temperature of 75°C for 8 min in an APAG machine.

#### 2.3.2 Therapeutic Method

CGF (180–200 pass) was injected face‐to‐face (Left/right face) via a hydrafacial injector (Derma shine, Panace Co. Ltd., Korea) for a total of three treatments at an interval of 1–2 months. Supramolecular salicylic acid (30%) (Broda, Bojinda Biochemical Technology Co. Ltd., Shanghai, China) was given immediately after each injection. Three treatments were given as one course of treatment. An amount of botulinum toxin (30–40 iu) was added to the first injection of CGF to compound the injection. Botulinum toxin was added to the first treatment only. Supramolecular salicylic acid operated by professional nurses: soap‐based cleansing followed by organic solvent degreasing, brush salicylic acid to test sensitivity for 3–5 min, massage to release to promote osmotic enhancement. Organic solvent degreasing to observe endpoint reaction: redness or redness + white cream, moist gauze to clean the face after cold compress or cold spray 20 min.

### 2.4 Observation Indicators

1. Comparison of clinical efficacy: The improvement rate of melasma area and severity index (MASI) score was used to evaluate the efficacy. The MASI score was used to evaluate the area, color depth, and uniformity of chloasma. The patient's face was divided into four regions, namely, the forehead, right cheek, left cheek, and mandible, with corresponding weights of 30%, 30%, 30%, and 10%, respectively. Cumulative area scores ranged from 0 to 6 points, and color depth and uniformity scores ranged from 0 to 4 points. The higher the MASI score, the severer the melasma. Basic cure was defined as a score reduction rate of at least 80%, and marked efficacy was defined as a score reduction rate of at least 50%. Effectiveness was defined as a score reduction rate of at least 10%. No effect was defined as the score reduction rate of less than 10%.
2. Skin image assessment: The VISIA digital skin analyzer was used to detect the skin of patients. The VISIA digital skin analyzer was used to detect the skin of patients. The measurements were carried out in a dark room protected from light, at room temperature of 20°C–26°C, and air relative humidity of 50% to 60%. Before the measurement, the patients had a rest of 15–20 min after cleansing the face with warm water and then underwent VISIA skin detection, and the area was analyzed to avoid interference factors such as nevus and hair. The main outcome measures included spots, wrinkles, texture, pores, UV spots, brown spots, red areas, and porphyrins.
3. Self‐evaluation of patient satisfaction: according to the improvement of patients' skin (including pigmentation, tightness, itching, and sting) before and after treatment, the evaluation results were divided into four grades, namely unsatisfactory, general, satisfied, and very satisfied, and the total satisfaction was calculated. Total satisfaction = (very satisfied+satisfied+general) number/total number of cases × 100%.

### 2.5 Statistical Methods

SPSS 27.0 software was used to process the data. The measurement data of normal or approximate distribution such as facial image analysis parameters were described by the mean ± standard deviation ($\bar{x}$ ± s). The comparison between the two groups was performed by paired sample t‐test or one‐way analysis of variance. The curative effect, type of melasma and other enumeration data were described by percentage (%) and compared by chi‐square test, Fisher's exact test or rank sum test.

## 3 Results

### 3.1 The Basic Information of the Included Subjects

Twenty female patients were included in this clinical trial, with the ages ranging from 25 to 45 years (average age of 41.3 years)，and the average disease duration was 4.5 years (ranged from 6 months to 10 years).

### 3.2 Results of Treatment Efficacy

**FIGURE 1:**
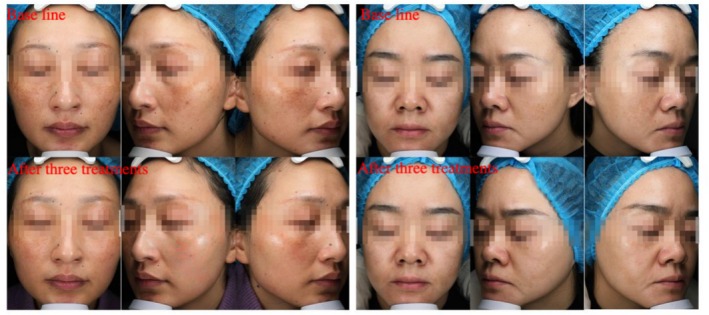
Pictures of typical cases before and after treatment.

The total effective rate of CGF combined with supramolecular salicylic acid in the treatment of melasma was 95%, of which 3 cases were basically cured, 14 cases were markedly effective, 2 cases were effective, and 1 case was ineffective (Table 1). The typical case is shown in Figure 1.

**TABLE 1:** Results of efficacy.

Indicators	After treatment (*n* = 20)
Basic cure	3 (15%)
Marked efficacy	14 (70%)
Effectiveness	2 (10%)
No effect	1 (5%)
Total effective rate	19 (95%)

### 3.3 Skin Image Assessment

The VISIA scores in spots, UV spots, brown spots, red areas, wrinkles, texture, pore, and porphyrins scores were significantly increased after treatment (*p* < 0.05, Table 2).

**TABLE 2:** Comparison of VISIA feature values before and after treatment.

Indicators	After treatment (*n* = 20)	Before treatment (*n* = 20)	t	P
Spots	51.52 ± 20.45	66.55 ± 16.12	2.581	0.014
UV spots	49.45 ± 30.10	66.15 ± 19.30	2.089	0.043
Brown spots	32.68 ± 24.03	57.30 ± 22.54	3.342	0.002
Red areas	34.92 ± 18.11	57.65 ± 20.90	3.676	< 0.001
Wrinkles	51.19 ± 23.14	71.90 ± 20.13	3.020	0.005
Texture	62.12 ± 25.40	77.40 ± 11.47	2.453	0.019
Pores	41.69 ± 35.57	62.55 ± 23.44	2.190	0.035
Porphyrins	73.22 ± 24.93	86.30 ± 8.18	0.886	0.032

### 3.4 Comparison of MASI Scores

**FIGURE 2:**
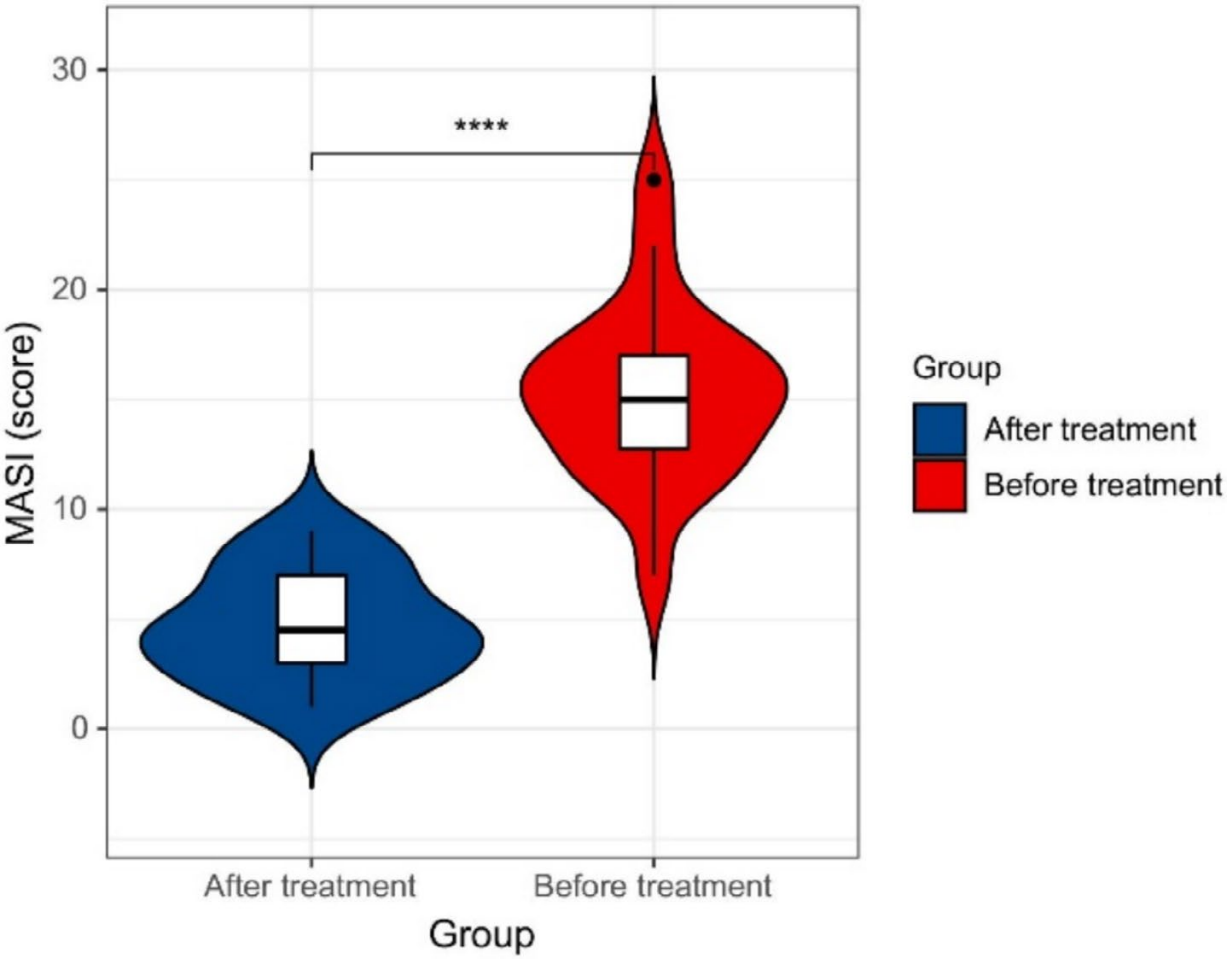
Comparison of MASI scores.

After CGF combined with supramolecular salicylic acid treatment, the MASI score of melasma patients was significantly reduced (4.70 ± 2.47 vs. 15.30 ± 4.01, *p* < 0.001, Table 3, Figure 2).

**TABLE 3:** Comparison of MASI scores.

Indicators	MASI score
After treatment (*n* = 20)	4.70 ± 2.47
Before treatment (*n* = 20)	15.30 ± 4.01
*t*	10.054
*p*	< 0.001

### 3.5 Results of Patient Satisfaction

The patients' satisfaction with the treatment effect was investigated by telephone follow‐up. The results showed that the total satisfaction of patients with facial skin improvement after CGF combined with supramolecular salicylic acid treatment was 95.00% (Table 4).

**TABLE 4:** Results of patient satisfaction.

Indicators	After treatment (*n* = 20)
Unsatisfactory	1 (5.00%)
Generally satisfactory	2 (10.00%)
Satisfied	10 (50.00%)
Very satisfied	7 (35.00%)
Total satisfaction	19 (95.00%)

### 3.6 Adverse Reactions

A few people reported mild pain during microneedling procedures, and it can be alleviated without special treatment. No skin adverse reactions related to the drug were observed.

## 4 Discussion

Chloasma, also known as liver spot, can occur in both men and women, most commonly in young and middle‐aged women, especially in Asian and African‐American populations [22]. Currently, the pathogenesis of chloasma has not been fully clarified. Family history of melasma, excessive sun exposure, pregnancy, cosmetic abuse, oral contraceptive pills or hormone replacement therapy, and certain chronic diseases, such as liver disease, tuberculosis, and autoimmune thyroid disease, are considered to be the triggering factors for the onset of chloasma [23]. The onset of chloasma is symmetrical and cyclical, with heavy onset in spring and summer, decreasing in autumn and winter, and the condition is persistent, difficult to treat, and easy to recur. The basic pathological change of melasma is melanin deposition. With the deepening of research, it is found that melanin deposition is not only limited to the epidermis but may even reach the dermis, which is also the reason for the recurrence of melasma. Compared with normal skin, the integrity of the stratum corneum and the basal layer of the skin is damaged in melasma, which leads to the descent of melanocytes and melanin into the dermis, deepening the depth of melanin deposition [24]. In turn, inflammatory cells in the dermis can enter the epidermis through the incomplete basal layer, aggravating the skin damage through inflammatory reaction and further promoting melanin deposition [25]. Therefore, the drugs commonly used in the clinic, whether oral drugs or topical drugs, cannot effectively solve the problem of recurrent chloasma if they only promote melanin decolouration and accelerate melanin metabolism. Current patient compliance favors external treatments, and oral medications are accompanied by significant side effects that many patients are reluctant to take orally. CGF combined with supramolecular salicylic acid treatment for refractory pigment diseases has the advantage of addressing the obvious window for skin pigment problems while also tackling the inflammation repair, skin photoaging, skin barrier decline, and other comprehensive problems, especially for the inability to assess whether the chloasma is advanced. The development period is more stable than the photoelectric treatment and is not prone to complications. Compared with photoelectric therapy, this combination method is safer, more stable, and more effective.

In this study, CGF combined with supramolecular salicylic acid was selected for the treatment of melasma. Pigment management is generally based on the whole skin layer, with CGF targeting intradermal pigments and supramolecular salicylic acid targeting epidermal pigments while penetrating into the CGF‐treated skin channels to promote pigment transport. Our results showed that the total effective rate of combined facial treatment was 95%, of which 15% were basically cured, 70% had a significant effect, 10% were effective, and only 5% of the patients showed no effect. The MASI scores of patients decreased significantly after treatment (4.70 ± 2.47 vs. 15.30 ± 4.01, *p* < 0.001). Patient satisfaction after treatment was 95.00%. CGF is prepared by dedicated special equipment. The main components are fibrous mesh scaffolds formed by abundant platelets, leukocytes, peripheral blood stem cells, and high concentrations of various growth factors and fibrinogen [26]. Previous study has shown PRP to be used as a stand‐alone treatment for melasma [27]. CGF is the newest blood concentrate containing growth factors, especially TGF‐β, which is one of the main growth factors used in the treatment of melasma [24]. TGF‐β reduces the signaling of microphthalmia‐inducing transcription factors, which in turn reduces tyrosinase and tyrosinase‐associated proteins [28]. In addition to this, CGF has the added benefit of inducing collagen synthesis, thereby improving the quality and texture of the skin [29]. In this study, botulinum toxin was compounded in CGF in the first treatment. The main purpose of botulinum toxin compounding in CGF is to improve the patient's skin in terms of vascularization and skin aging. Botulinum toxin indirectly affects the melanogenesis process by inhibiting pro‐inflammatory cytokines mediated by the interaction of melanocytes, keratinocytes and fibroblasts [30]. Supramolecular technology allows for the selective formation of molecule‐to‐molecule supramolecular aggregates of non‐water‐soluble salicylic acid molecules with other molecules in a molecularly recognizable and self‐assembling manner, without the addition of organic solvents and alkaline neutralizing agents. This new supramolecular salicylic acid forms water‐soluble supramolecular salicylic acid composite aggregates through intermolecular reversible, non‐covalent bonding interactions [31]. Salicylic acid can be released slowly and controllably by reversible dissociation of salicylic acid molecules through hydrogen bonding in water, which not only gives full play to the efficacy of salicylic acid but also significantly reduces the side effects. The treatment of melasma with 30% supramolecular salicylic acid resulted in a significant decrease in MASI scores compared to baseline values, which is consistent with our results [32].

Facial spots, UV spots, brown spots, red zones, wrinkles, texture, pore, and porphyrins scores all increased significantly after treatment as measured by VISIA. The red zone can reflect facial inflammation [33]. This result also showed a significant reduction in facial inflammatory response. In this study, the treated CGF was able to enrich CD34+ cells in the blood, effectively sorting out the important hematopoietic stem cell (HSC)/hematopoietic progenitor cell (HPC), and replenishing various blood cell components at the injection site [34]. At the same time, CD34+ molecules can activate vascular endothelial cells, which migrate under the action of adhesion molecules and chemokines, facilitating endothelial repair and revascularization [35]. The improved blood supply to the face promotes the proliferation of fibroblasts, secretory cells, and the production of collagen fibers, and the repair of the vascular endothelium reduces the exudation of inflammatory cells, further controlling the development of inflammation [36]. The restoration of blood supply and the reduction of inflammation around the skin appendages improve the secretion function of the glands, reduce the symptoms of dry and rough skin, enhance blood circulation, accelerate the metabolism of melanocytes in the dermis, and reduce pigmentation, which is manifested in the reduction of spots and increase in luster [37]. At the same time, the accelerated renewal of epidermal cells enables the timely elimination of oil from the pores, which is reflected in the increase of pore score. fibrin, fibronectin, and pro‐phospholipidic adhesion protein contained in CGF gather the growth factors released by platelets in the local area. CGF can also act as a matrix to provide a favorable place for cell attachment, migration, and differentiation, and become a scaffold for new cells and tissues to promote the repair of aged skin, increase skin luster, and restore the elasticity and fullness of the skin, which is manifested as an increase in the wrinkle and texture scores [38]. Supramolecular salicylic acid is given immediately after CGF injection to further inhibit melanin production and counteract the inflammatory response in chloasma patients [39].

In conclusion, CGF combined with cyclic injection of supramolecular salicylic acid is a new combined cosmetic technology, and there is no case of this combination at present. The results of this study show that this combination program is effective in the treatment of melasma, with high patient satisfaction, safety, and no stimulation. However, due to the limited duration of treatment in this trial, it is necessary to further extend the course of treatment to observe the recurrence of chloasma. The sample size of this study was small, and the gender was female; the study only evaluated the therapeutic effect of CGF combined with 30% supramolecular salicylic acid on melasma through self‐parallel control and did not investigate the difference in the effect with other therapeutic means, such as topical or systemic medication and laser treatment; the duration of the trial was only 1 cycle, which could not reflect the therapeutic effect of the treatment of recurrent melasma lesions, and therefore further studies are needed to be conducted to determine the effectiveness of the treatment. Therefore, further studies are needed.

## 5 Conclusion

The therapeutic effect of CGF combined with 30% supramolecular salicylic acid on melasma is remarkable, which significantly improves the skin quality without complications, is safe and reliable, and has high patient satisfaction, which is worthy of being further promoted and applied as a new idea for the treatment of melasma in the clinic.

## Author Contributions

Ge Su and Qiang Fu conceptualized and designed the study, and them as the co‐first authors. Ge Su collected the data and drafted the manuscript, Qiang Fu analyzed the data. Xun Zhou edited this manuscript as a native. All authors read and approved the final manuscript.

## Ethics Statement

The study was conducted in accordance with the Declaration of helsinki and approved by the Ethics Committee of Chongqing Hospital of Traditional Chinese Medicine (CQHTCM). All data in this article are represented in graphical form in the article or in supplementary documents.

## Conflicts of Interest

The authors declare no conflicts of interest.

## Data Availability

The data that support the findings of this study are available on request from the corresponding author. The data are not publicly available due to privacy or ethical restrictions.

## References

[1] Q. Chen , L. Liu , and Y. Zhang , “Vitamin D and Wound Healing: Assessing Skin Barrier Function and Implications for Chloasma Treatment,” International Wound Journal 21, no. 5 (2024): 14893, 10.1111/iwj.14893. PMC10789544 38272820

[2] L. J. Wang , Y. B. Pang , W. Q. Li , et al., “Global Research Trends on Melasma: A Bibliometric and Visualized Study From 2014 to 2023,” Frontiers in Pharmacology 15 (2024): 1421499, 10.3389/fphar.2024.1421499. 39119611 PMC11306164

[3] Y. T. He , Y. Y. Hao , R. X. Yu , et al., “Hydroquinone Cream‐Based Polymer Microneedle Roller for the Combined Treatment of Large‐Area Chloasma,” European Journal of Pharmaceutics and Biopharmaceutics 185 (2023): 5–12, 10.1016/j.ejpb.2023.01.024. 36739099

[4] D. Morgado‐Carrasco , J. Delgado , L. Prudkin‐Silva , J. Santamaria , and J. Piquero‐Casals , “Sunscreens Prescribed to Patients With Skin of Color and/or With Melasma: A Survey of 221 Dermatologists and Dermatology Residents in Spain,” Photodermatology, Photoimmunology & Photomedicine 40, no. 5 (2024): e12996, 10.1111/phpp.12996. 39149878

[5] Y. Y. Chen , Y. P. Bai , and W. H. Duan , “Identification and Analysis of Chloasma Based on Blood Collateral Theory,” Beijing Journal of Traditional Chinese Medicine 42, no. 10 (2023): 1104–1107, 10.16025/j.1674-1307.2023.10.013.

[6] J. Wan , Z. Liao , B. Dong , S. Jiang , and T. Lei , “Targeting Senescent Dermal Fibroblasts Responsible for Hyperactive Melanocytes in Melasma,” Chinese Medical Journal 136, no. 13 (2023): 1563–1565, 10.1097/CM9.0000000000002488. 37057732 PMC10325758

[7] S. Zhao , M. Wang , X. Lai , and Y. Yan , “Efficacy and Safety of Ablative Fractional Laser in Melasma: A Meta‐Analysis and Systematic Review,” Lasers in Medical Science 39, no. 1 (2024): 71, 10.1007/s10103-024-03972-w. 38379033

[8] O. Artzi , T. Horovitz , E. Bar‐Ilan , et al., “The Pathogenesis of Melasma and Implications for Treatment,” Journal of Cosmetic Dermatology 20, no. 11 (2021): 3432–3445, 10.1111/jocd.14382. 34411403

[9] R. Liang , H. Luo , W. Pan , et al., “Comparative Efficacy and Safety of Tranexamic Acid for Melasma by Different Administration Methods: A Systematic Review and Network Meta‐Analysis,” Journal of Cosmetic Dermatology 23, no. 4 (2024): 1150–1164, 10.1111/jocd.16104. 38059683

[10] X. Li , L. Chen , H. Wang , Y. Li , H. Wu , and F. Guo , “Germacrone, Isolated From Curcuma Wenyujin, Inhibits Melanin Synthesis Through the Regulation of the MAPK Signaling Pathway,” Journal of Natural Medicines 78, no. 4 (2024): 863–875, 10.1007/s11418-024-01818-x. 38809333

[11] V. González‐Molina , A. Martí‐Pineda , and N. González , “Topical Treatments for Melasma and Their Mechanism of Action,” Journal of Clinical and Aesthetic Dermatology 15, no. 5 (2022): 19–28. PMC9122278 35642229

[12] S. Singh , M. K. Singh , and P. Das , “Visual Detection of Cyclobutane Pyrimidine Dimer DNA Damage Lesions by Hg2+ and Carbon Dots,” Analytica Chimica Acta 1016 (2018): 49–58, 10.1016/j.aca.2018.02.029. 29534804

[13] E. Fitsiou , T. Pulido , J. Campisi , F. Alimirah , and M. Demaria , “Cellular Senescence and the Senescence‐Associated Secretory Phenotype as Drivers of Skin Photoaging,” Journal of Investigative Dermatology 141, no. 4S (2021): 1119–1126, 10.1016/j.jid.2020.09.031. 33349436

[14] A. Salminen , K. Kaarniranta , and A. Kauppinen , “Photoaging: UV Radiation‐Induced Inflammation and Immunosuppression Accelerate the Aging Process in the Skin,” Inflammation Research 71, no. 7–8 (2022): 817–831, 10.1007/s00011-022-01598-8. 35748903 PMC9307547

[15] A. N. Sharma , C. M. Kincaid , and N. A. Mesinkovska , “The Burden of Melasma: Race, Ethnicity, and Comorbidities,” Journal of Drugs in Dermatology 23, no. 8 (2024): 691–693, 10.36849/JDD.8233. 39093647

[16] A. T. Abdel‐Rahman , F. G. Abdel‐Hakeem , and M. H. Ragaie , “Clinical, Dermoscopic, and Histopathologic Evaluation of Vitamin C Versus PRP, With Microneedling in the Treatment of Mixed Melasma: A Split‐Face, Comparative Study,” Dermatologic Therapy 35, no. 2 (2022): e15239, 10.1111/dth.15239. 34851010

[17] L. C. Santos , G. L. Lana , G. S. Santos , et al., “The Biological Role of Platelet Derivatives in Regenerative Aesthetics,” International Journal of Molecular Sciences 25, no. 11 (2024): 5604, 10.3390/ijms25115604. 38891792 PMC11172268

[18] N. Atsu , C. Ekinci‐Aslanoglu , and B. Kantarci‐Demirkiran , “The Comparison of Platelet‐Rich Plasma Versus Injectable Platelet Rich Fibrin in Facial Skin Rejuvenation,” Dermatologic Therapy 2023, no. 1 (2023): 3096698, 10.1155/2023/3096698.

[19] B. Knoll and B. Hersant , “Invited Discussion on: Concentrated Growth Factor (CGF): The Newest Platelet Concentrate and Its Application in Nasal Hyaluronic Acid Injection Complications,” Aesthetic Plastic Surgery 47, no. 5 (2023): 1794–1795, 10.1007/s00266-023-03359-2. 37145318

[20] L. Lei , Y. Yu , J. Han , et al., “Quantification of Growth Factors in Advanced Platelet‐Rich Fibrin and Concentrated Growth Factors and Their Clinical Efficacy as Adjunctive to the GTR Procedure in Periodontal Intrabony Defects,” Journal of Periodontology 91, no. 4 (2020): 462–472, 10.1002/JPER.19-0290. 31471902

[21] E. B. Friedman , R. A. Scolyer , and J. F. Thompson , “Management of Pigmented Skin Lesions During Pregnancy,” Australian Journal of General Practice 48, no. 9 (2019): 621–624, 10.31128/AJGP-04-19-48952. 31476830

[22] A. C. C. Espósito , D. P. Cassiano , C. N. da Silva , et al., “Update on Melasma‐Part I: Pathogenesis,” Dermatologic Therapy 12, no. 9 (2022): 1967–1988, 10.1007/s13555-022-00779-x. PMC9464278 35904706

[23] Z. Jia , K. Tian , Y. Zhong , et al., “Effectiveness of Combination Therapy of Broadband Light and Intradermal Injection of Tranexamic Acid in the Treatment of Chloasma,” Journal of Cosmetic Dermatology 22, no. 5 (2023): 1536–1544, 10.1111/jocd.15632. 36718828

[24] T. R. Galache , M. M. Sena , J. A. F. Tassinary , and C. Pavani , “Photobiomodulation for Melasma Treatment: Integrative Review and State of the Art,” Photodermatology, Photoimmunology & Photomedicine 40, no. 1 (2024): e12935, 10.1111/phpp.12935. 38018017

[25] W. Liu , Q. Chen , and Y. Xia , “New Mechanistic Insights of Melasma,” Clinical, Cosmetic and Investigational Dermatology 16 (2023): 429–442, 10.2147/CCID.S396272. 36817641 PMC9936885

[26] P. C. Tan , P. Q. Zhang , S. B. Zhou , et al., “Autologous Concentrated Growth Factor Increases Skin Thickness and Area During Tissue Expansion: A Randomized Clinical Trial,” Plastic and Reconstructive Surgery 152, no. 2 (2023): 281e–292e, 10.1097/PRS.0000000000010227.36727707

[27] Y. Kashikar , B. Madke , A. Singh , S. Meghe , and K. Rusia , “Mesotherapy for Melasma – An Updated Review,” Journal of Pharmacy & Bioallied Sciences 16, no. Suppl 2 (2024): S1055–S1056, 10.4103/jpbs.jpbs_1192_23. 38882767 PMC11174183

[28] L. Zhao , M. Hu , Q. Xiao , et al., “Efficacy and Safety of Platelet‐Rich Plasma in Melasma: A Systematic Review and Meta‐Analysis,” Dermatologic Therapy 11, no. 5 (2021): 1587–1597, 10.1007/s13555-021-00575-z. PMC8484406 34269967

[29] R. Zhou , M. Wang , X. Zhang , et al., “Therapeutic Effect of Concentrated Growth Factor Preparation on Skin Photoaging in a Mouse Model,” Journal of International Medical Research 48, no. 10 (2020): 300060520962946, 10.1177/0300060520962946. 33115316 PMC7645418

[30] J. A. Jung , B. J. Kim , M. S. Kim , et al., “Protective Effect of Botulinum Toxin Against Ultraviolet‐Induced Skin Pigmentation,” Plastic and Reconstructive Surgery 144, no. 2 (2019): 347–356, 10.1097/PRS.0000000000005838. 31348342

[31] Y. Cheng , L. Zhang , and Y. You , “The Effects of Supramolecular Nicotinamide Combined With Supramolecular Salicylic Acid on Chloasma,” Journal of Cosmetic Dermatology 23, no. 2 (2024): 681–686, 10.1111/jocd.16010. 38111320

[32] Y. Xiong , X. Jiang , W. Lai , et al., “Supramolecular Salicylic Acid Combined With Niacinamide in Chloasma: A Randomized Controlled Trial,” Clinical and Experimental Dermatology 49, no. 11 (2024): 1330–1337, 10.1093/ced/llae135. 38618759

[33] L. Ma , X. Huang , Y. Qiu , and Y. He , “Analysis of Facial Redness by Comparing VISIA and YLGTD,” Skin Research and Technology 29, no. 7 (2023): e13356, 10.1111/srt.13356. 37522504 PMC10280608

[34] J. Gao , Q. Xiao , Y. Lu , et al., “Higher Percentage of CD34+ Stem Cells and Elevated Efficacy in Androgenetic Alopecia Treatment Observed in CGF Prepared From 640 Nm Laser‐Pretreated Blood: A Preliminary Study,” Journal of Cosmetic Dermatology 23, no. 6 (2024): 2249–2255, 10.1111/jocd.16249. 38429917

[35] P. Pederzoli , A. Greco Lucchina , M. Del Fabbro , and C. Mortellaro , “Concentrated Growth Factors Gel Activated With Ozone for Facial Aesthetics Purpose After Granuloma Removal: A Case Report,” Journal of Biological Regulators and Homeostatic Agents 35, no. 2 Suppl. 1 (2021): 345–350, 10.23812/21-2supp1-34. 34281331

[36] L. Huang , Y. Dong , C. Li , S. Han , and B. Cheng , “Effect of Platelet Concentrate Prepared by Different Methods on the Healing of Full‐Thickness Skin Defects,” Journal of Cosmetic Dermatology 21, no. 11 (2022): 5910–5921, 10.1111/jocd.15204. 35778915

[37] G. Li and H. Wang , “Novel Applications of Concentrated Growth Factors in Facial Rejuvenation and Plastic Surgery,” Facial Plastic Surgery 40, no. 1 (2024): 112–119, 10.1055/a-1987-3459. 36423628

[38] R. Pensato , R. Al‐Amer , and S. La Padula , “A Comprehensive Review of Concentrated Growth Factors and Their Novel Applications in Facial Reconstructive and Regenerative Medicine,” Aesthetic Plastic Surgery 48, no. 15 (2024): 3022–3023, 10.1007/s00266-023-03539-0. 37580569

[39] X. Shao , Y. Chen , L. Zhang , et al., “Effect of 30% Supramolecular Salicylic Acid Peel on Skin Microbiota and Inflammation in Patients With Moderate‐To‐Severe Acne Vulgaris,” Dermatologic Therapy 13, no. 1 (2023): 155–168, 10.1007/s13555-022-00844-5. PMC9823178 36350527

